# Optical Sensors and Actuators for Probing Proximity-Dependent Biotinylation in Living Cells

**DOI:** 10.3389/fncel.2022.801644

**Published:** 2022-02-16

**Authors:** Rui Chen, Ningxia Zhang, Yubin Zhou, Ji Jing

**Affiliations:** ^1^Department of Oral and Maxillofacial Surgery, Sun Yat-sen Memorial Hospital, Sun Yat-sen University, Guangzhou, China; ^2^Laboratory of Cancer Biology, Department of Medical Oncology, Institute of Clinical Science, Sir Run Run Shaw Hospital, College of Medicine, Zhejiang University, Hangzhou, China; ^3^Department of Translational Medical Sciences, Center for Translational Cancer Research, Institute of Biosciences and Technology, College of Medicine, Texas A&M University, Houston, TX, United States; ^4^The Cancer Hospital of the University of Chinese Academy of Sciences (Zhejiang Cancer Hospital), Institute of Basic Medicine and Cancer, Chinese Academy of Sciences, Hangzhou, China

**Keywords:** proximity labeling, TurboID, optogenetics, OptoID, biotinylation

## Abstract

Proximity-dependent biotinylation techniques have been gaining wide applications in the systematic analysis of protein-protein interactions (PPIs) on a proteome-wide scale in living cells. The engineered biotin ligase TurboID is among the most widely adopted given its enhanced biotinylation efficiency, but it faces the background biotinylation complication that might confound proteomic data interpretation. To address this issue, we report herein a set of split TurboID variants that can be reversibly assembled by using light (designated “OptoID”), which enable optogenetic control of biotinylation based proximity labeling in living cells. OptoID could be further coupled with an engineered monomeric streptavidin that permits real-time monitoring of biotinylation with high temporal precision. These optical actuators and sensors will likely find broad applications in precise proximity proteomics and rapid detection of biotinylation in living cells.

## Introduction

Proximity labeling (PL) has been gaining more and more applications to identify protein-protein interactions (PPIs) on a proteome-wide scale under more physiologically relevant conditions within living cells (Qin et al., 2021). Promiscuous enzymes, such as proximity-dependent biotin identification (BioID) (Choi-Rhee et al., 2004; Roux et al., 2012) and ascorbic acid peroxidase (APEX) (Rhee et al., 2013; Lam et al., 2015), have been engineered to label endogenous proteins within a few nanometers. APEX is derived from ascorbate peroxidase that catalyzes the oxidation of biotin-phenol to the short-lived (<1 ms) biotin-phenoxyl radical in the presence of hydrogen peroxide (H_2_O_2_) (Rhee et al., 2013; Lam et al., 2015). By contrast, a biotin ligase, such as BioID derived from *Escherichia coli* biotin ligase (BirA), does not require reactive reagents, but simply utilizes the highly soluble biotin as a more cell-friendly substrate (Choi-Rhee et al., 2004; Roux et al., 2012; Qin et al., 2021). BirA specifically binds and biotinylates the biotin carboxyl carrier protein subunit of acetyl-CoA carboxylase by forming an amide bond between a reactive biotin and a specific lysine residue on the protein surface. BioID, a mutant variant of BirA (R118G), shows lower affinity for the biotinoyl-adenylate (bioAMP), which leads to the premature release of the reactive bioAMP from the catalytic site, allowing the promiscuous biotinylation of proximate proteins (Choi-Rhee et al., 2004). However, the prototypical version of BioID has a low activity (>18 h of labeling time) (Choi-Rhee et al., 2004; Roux et al., 2012; Qin et al., 2021). Recently, directed-evolution variants of BioID, namely the TurboID and the miniTurbo, have been developed, with both showing rapid biotinylation kinetics and gaining wider applications in many biological processes (Branon et al., 2018). TurboID, nonetheless, is known to exhibit relatively high background biotinylation to confound biotin labeling and subsequent proteomic studies (Branon et al., 2018; Cho et al., 2020). To address this limitation, researchers have screened and identified an optimal site to split the TurboID into two inactive fragments. The split TurboID restores its function when the two parts are brought by using a rapamycin-dependent chemically induced dimerization system (Cho et al., 2020). However, such chemogenetic approach lacks sufficient spatial resolution (Table 1). Furthermore, rapamycin tends to engage endogenous mTOR signaling to perturb the host physiology. Improved tools with both spatial and temporal controllability are needed to enable precise proximity biotinylation.

**TABLE 1:** Comparison between OptoID and existing split biotin ligases (BirA) and TurboID systems based on chemical-inducible dimerization (CID).

Tools	Engineering targets	Prediction templates	Prediction strategy	Split site	Dimerizer	Trigger	Attributes	References
OptoID	TurboID	PDB: 1BIB	SPELL	G99/E100	iLID	Blue light	Temporal and spatial control; least invasiveness; reduced cytotoxicity	This study
Split-BioID	BioID	PDB: 1BIB	Structural analysis	E256/G257	FKBP/FRB	Rapamycin	Retains temporal control but without spatial resolution; Potential toxicity and signaling cross-talk	Schopp et al., 2017
Contact-ID	BioID	PDB: 1HXD	B factor	G78/G79	FKBP/FRB	Rapamycin		Kwak et al., 2020
Split-TurboID	TurboID	PDB: 1HXD and 2EWN	SPELL	L73/G74	FKBP/FRB	Rapamycin		Cho et al., 2020
Split-BioID	BirA*	PDB: 1BIB	Structural analysis	E140/Q141	PP1/PIP	PPI	Identifies vicinal proteins closely positioned in space; lack of temporal and spatial control	De Munter et al., 2017

SPELL, split proteins reassembly by a ligand or by light; iLID, improved light-induced dimer; B Factor, temperature factors; FKBP, 12-KDa FK506-binding protein; FRB, FKBP-rapamycin-binding domain; PP1, protein phosphatase; PIP, PP1-interacting protein; PPI, protein-protein interaction; BirA*, BirA R118G.

Another tool that is equally important for probing proximity proteomics is a genetically encoded sensor to report biotinylation at real time in living cells. Monomeric Streptavidin (mSA) is an engineered streptavidin that binds biotin and biotinylated proteins with a high affinity (Lim et al., 2011). mSA facilitates the imaging and tracking of biotinylation in live cells. However, we found that when expressed in mammalian cells such as HeLa cells, mSA-EGFP exhibited a high tendency to form aggregates, making it less ideal as a molecular probe for biotinylation. In the current study, we report a mSA (K56R) mutant, which showed less propensity to form aggregates. This engineered biotinylation biosenor permits real-time monitoring of biotinylation with high temporal precision. Furthermore, in order to reduce the background biotinylation activity of TurboID, we designed a photo-sensitive split TurboID (termed as “OptoID”) that can be assembled by using light, which enables temporal and spatial control of biotinylation based proximity labeling.

## Materials and Methods

### Antibodies and Reagents

Streptavidin-HRP [N100, 1:4,000 for western blotting (WB)] and Dynabeads MyOne Streptavidin C1 (#65001) were purchased from Thermo Fisher Scientific. Rabbit anti-biotin antibody (ICP0611) was purchased from ImmuneChem. An anti-mCherry antibody was obtained from Novus Biologics (NBP2-25157). A mouse monoclonal anti-Flag antibody (no. F3165) was purchased from Sigma. Secondary antibody for immunofluorescence staining, anti-rabbit Alexa fluor 488, was obtained from molecular probes (1:1,000). Secondary antibodies for western blot, goat anti-rabbit IgG-HRP (sc-2004, 1:2,000) and goat anti-mouse IgG-HRP (sc-2005), were purchased from Santa Cruz Biotechnology. Enhanced chemiluminescence (ECL) western blotting substrate was obtained from Thermo Fisher Scientific (#32106). KOD Hot Start DNA polymerase (#71086-4) was purchased from Sigma. The T4 DNA ligase kit (#M0202M) and NEBuilder HiFi DNA Assembly Master Mix (M5520AA) were purchased from New England BioLabs.

### Plasmids

mSA-EGFP was purchased from Addgene (#39863). For the STIM1-mCherry construct, full-length cDNA of human STIM1 was sub-cloned into mCherry-N1 (#54517, Addgene) between *Nhe*I and *Xho*I. STIM1-TurboID-mCherry was made by inserting TurboID (#107171, Addgene) into STIM1-mCherry between STIM1 and mCherry by using the *Hin*dIII and *Age*I restriction sites. To generate STIM1-OptoID-mCherry, cDNAs encoding the “iLID-P2A-SspB” element was synthesized as a gBlock and then inserted into STIM1-TurboID-mCherry at the selected insertion sites by using the NEBuilder HiFi DNA Assembly Master Mix.

### Cell Culture and Transfection

HeLa (CCL-2) was purchased from American Type Culture Collection (ATCC), with the identity validated by short tandem repeat profiling analysis by the vendor. Cells were free of mycoplasma contamination. HeLa cells were routinely cultured in Dulbecco’s modified Eagle’s medium (DMEM, Sigma) supplemented with 100 units/ml penicillin, 100 μg/ml streptomycin, and 10% fetal bovine serum (FBS) and grown in a 37°C humidified incubator containing 5% CO_2_.

### Immunoblotting

The transfected cells were washed in chilled PBS three times and lysed directly using cell lysis buffer (#9803, Cell Signaling Technology), including 20 mM Tris–HCl (pH 7.5), 150 mM NaCl, 1 mM EDTA, 1 mM EGTA, 1% Triton X-100, 2.5 mM Na pyrophosphate, 1 mM β-glycerophosphate, pH7.5 for 30 min at 4°C. The lysis buffer contained 1X protease inhibitor cocktail (P3100-010, GenDEPOT) and phosphatase inhibitor cocktail (P3200-001, GenDEPOT) to prevent degradation and removal of post-translational modifications. The lysates were clarified by centrifugation at 20,000 *g* at 4°C for 10 min. The supernatant was collected, with the total protein concentrations determined by a BCA protein assay (#23225; Thermo Scientific). For immunoprecipitation experiments, equivalent sample amounts were incubated with the magnetic Streptavidin C1 beads (#65001, Thermo Scientific) overnight at 4°C. The beads were then pelleted and washed with lysis buffer five times. 1X denaturing loading buffer was added and heated for 5 min at 95°C before loading into SDS-PAGE. Cell lysates were electrophoretically separated on 8–16% SDS-PAGE (M00660, GenScript), followed by transferring to PVDF membranes and probing with appropriate antibodies.

### Fluorescence Imaging

HeLa cells were cultured on 35-mm glass-bottomed dishes (#D35-20-0-TOP, Cellvis) at 37°C with 5% CO_2_. The cells were then transfected with the indicated plasmids by using the Lipofectamine 3000 (Invitrogen) transfection kit according to the manufacturer’s instructions. After transfection for 24 h, samples were mounted on a Nikon Ti2 Inverted microscope equipped with a Yokogawa W-1 dual spinning disk scanhead and Micro-Scanner for photo-stimulation and a stage top incubator for live cell imaging. The captured images were analyzed by the NIS-Elements AR microscope imaging software (Nikon NIS-element AR version 4.0).

### Real-Time Intracellular Ca^2+^ Measurement

For measurements of store-operated Ca^2+^ entry using Fluo-4 AM dye (Thermo Fisher Scientific), HeLa cells transfected with STIM1-TurboID-mCherry were incubated with 5 μM Fluo-4 for 25–30 min at 37°C and kept in a Ca^2+^ free solution. 1 μM thapsigargin was used to induce ER Ca^2+^ depletion. After depletion, the incubator buffer was switched to a 2 mM Ca^2+^ extracellular buffer. Fluorescence imaging was recorded on a Nikon Ti2.

## Results

### mSA-EGFP for Real-Time Monitoring of TurboID-Mediated Biotinylation

**FIGURE 1:**
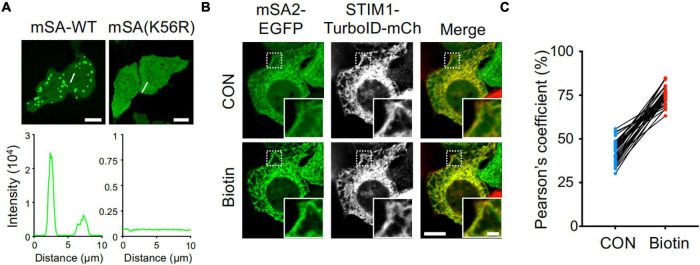
High background activity of TurboID revealed by mSA-EGFP labeling of biotinylation. **(A)** Representative confocal images of HeLa cells expressing mSA-WT-EGFP and K56R mutant. Scale bar, 10 μm. **(B)** Images of HeLa cells co-expressing mSA (K56R; named as mSA2)-EGFP and STIM1-TurboID-mCherry (mCh) with or without biotin treatment (100 μM). Scale bar, 10 μm. Insets, zoomed-in views of boxed regions. Scale bar, 2 μm. The mCherry-channel images are shown in grayscale. Also see Supplementary Movie 1. **(C)** Pearson’s correlation coefficient to quantify the colocalization between mSA2-EGFP and STIM1-TurboID-mCherry (*n* = 30 cells from three biological replicates).

To monitor the biotin labeling process catalyzed by TurboID in living cells, we utilized an engineered monomeric streptavidin (mSA) tagged with enhanced green fluorescent protein (EGFP). mSA has been shown to tightly bind biotin with a binding affinity of 2.8 nM *in vitro* (Lim et al., 2013). However, when expressed in mammalian cells such as HeLa cells, mSA-EGFP exhibited a high tendency to form aggregates (Figure 1A and Supplementary Figure 1A), making it less ideal as a molecular probe for biotinylation. Previous studies have shown that by replacing short interfacial residues (A72, G74, T76, A89, T91, and S93) of streptavidin with bulky charged residues (K72, E74, R76, R89, E91, and R93) could yield a stable monomeric streptavidin (Lim et al., 2011). These residues were targeted because small amino acids, are typically poor β-sheet formers, whereas large residues and branched amino acids can use their bulky side chains to shield the main chain from the bulk solvent and thus stabilize the main chain hydrogen bonds (Lim et al., 2011). Further replacement of the biotin binding residues of streptavidin with the biotin binding residues of rhizavidin led to the design of a high affinity monomeric variant of streptavidin (Lim et al., 2013). The K56 of mSA is corresponding to the A72 of streptavidin (Lim et al., 2013). Interestingly, we found that when K56 was further mutated to arginine (K56R), this mutant showed a reduction in aggregate formation in the cells (Figure 1A). Because mSA (K56R; named as mSA2)-EGFP is less prone to form appreciable aggregates, we decided to use this construct as the biosensor in the following studies.

To aid real-time visualization and analysis of biotinylation, we next used an ER-resident protein, the stromal interaction molecular 1 (STIM1) involved in Ca^2+^ signaling at inter-organellar membrane contact sites (Nguyen et al., 2018), as our test case. TurboID was fused to mCherry-tagged STIM1 to yield ER-bound STIM1-TurboID-mCherry (Figure 1B and Supplementary Figure 1A). The function of STIM1 is not altered after fusing with TurboID and mCherry. This construct was correctly localized to endoplasmic reticulum (ER, Figure 1B and Supplementary Figure 1A), and exhibited integrated store-operated Ca^2+^ entry (SOCE) kinetics (Supplementary Figure 1B). When co-expressed STIM1-TurboID-mCherry and mSA2-EGFP in HeLa cells in the absence of externally added biotin, we noted prominent ER-like distribution of mSA2-EGFP that tightly colocalized with STIM1-TurboID-mCherry (Figure 1B and Supplementary Movie 1). As control, mSA2-EGFP showed an even distributed in cells co-overexpressing STIM1-mCherry before biotin treatment (Supplementary Figure 1C). Upon biotin treatment, more mSA2-EGFP was found to co-localize with STIM1-TurboID-mCherry (Figures 1B,C), but not with STIM1-mCherry (Supplementary Figure 1C). The mean value of Pearson’s coefficient values was 43.03 ± 6.57% in the absence of biotin treatment (Figure 1C), indicating that TurboID has high background biotinylation activity even at the endogenous level of biotin.

### Design and Optimization of OptoID

**FIGURE 2:**
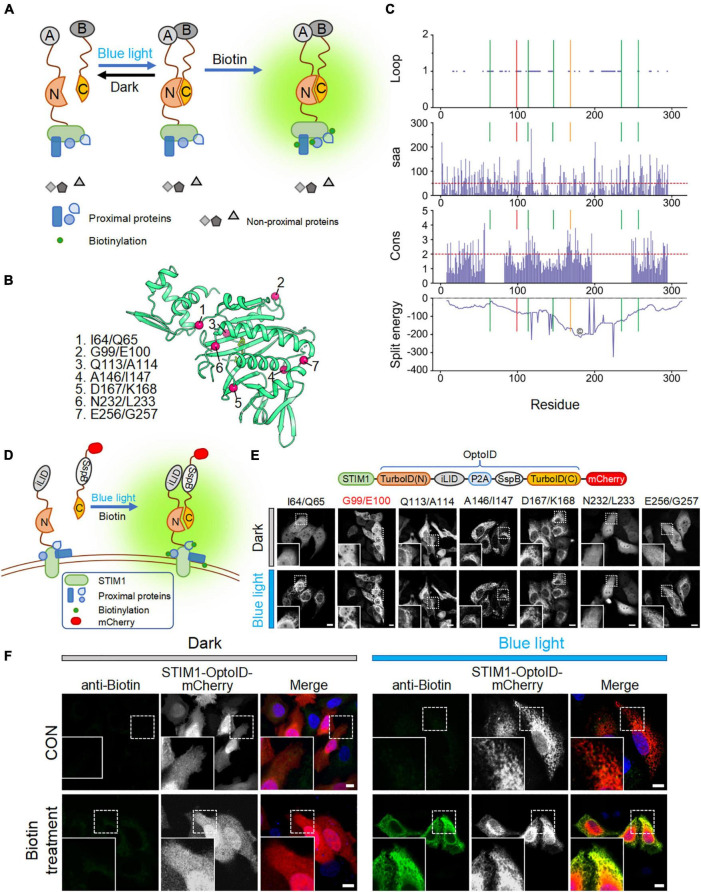
Design and optimization of OptoID. Photostimulation was applied with a 488-nm confocal laser (5% output, 1 s ON for each 5 s). Scale bar, 10 μm. The mCherry-channel images are shown in grayscale. **(A)** Design and mode of light–controlled re-assembling of split TurboID. A, iLID; B, SspB. N, N-terminal half of TurboID; and C, C-terminal half of TurboID. **(B)** The 3D structure of BirA (PDB entry: 1BIB) with putative split sites indicated by red spheres. **(C)** Seven split sites were predicted by the SPELL algorithm. Three parameters [including Loop, solvent accessible area (saa), and sequence conservation (cons)] and split energy were displayed in the bar and curve graph, respectively. *X* axis was shown residue number. Lines show the predicted split sites. Red Line is G99/I100 split site. Orange line is D167/K168 split site. The core (©) was described as split energy minima. **(D)** Schematic illustration of the design of a light-switchable split TurboID. iLID was fused to the N-terminal half of TurboID (N) conjugated with STIM1 and SspB was fused to the C-terminal half (C) followed by mCherry. A functional TurboID is re-assembled upon light stimulation to bring two parts in close proximity. **(E)** Diagram of the STIM1-OptoID-mCherry constructs (upper panel). Confocal images of HeLa cells expressing the indicated constructs before and after light stimulation (lower panel). The N terminal loop of TurboID is fused to ER-resident STIM1 and C terminal loop of TurboID tagged with mCherry. Two fragments are congregated by Light-inducible heterodimer (iLID-P2A-SspB) system. Upon light stimulation, functional reassembly of split-TurboID is anticipated to restore biotinylation activity with subsequent ER-like distribution of mCherry-tagged TurboID C terminal loop. Insets: zoomed-in views of boxed regions. **(F)** Confocal images of HeLa cells expressing STIM1-OptoID-mCherry (with iLID-P2A-SspB inserted at G99/E100) in response to blue light stimulation with or without biotin treatment (100 μM). Rabbit anti-biotin antibody and a secondary anti-rabbit Alexa Fluor Plus 488 (probe to biotin antibody) was used as an indicator for biotinylation. Blue, nuclear staining with Hoechst 33342.

To reduce the background biotinylation activity of TurboID, we set out to split TurboID into two non-functional fragments, and fused them with an optical dimerization system termed as iLID-SspB (Guntas et al., 2015). iLID contains a LOV2-caged SsrA peptide that could restore its interaction with its natural binding partner, SspB, in a blue light-dependent manner (Guntas et al., 2015). We anticipated that the enzymatic function of split-TurboID could be restored upon light-induced association between the two split parts, thereby conferring temporal and spatial control over TurboID-catalyzed biotinylation activity (Figure 2A).

We first used a previously reported algorithm (split proteins reassembly by a ligand or by light, SPELL) to predict candidate split sites through *silico* analysis (Dagliyan et al., 2018). The SPELL algorithm considers solvent accessibility, sequence conservation, and geometric constraints to evaluate potential split sites, and has the ability to pinpoint fragment pairs that give high reconstitution efficiency but minimal spontaneous assembly (Dagliyan et al., 2018; Cho et al., 2020). Because crystal structures for TurboID are not available, we applied the SPELL algorithm to wild-type *Escherichia coli* biotin ligase (BirA, PDB ID: 1BIB) (Wilson et al., 1992). Indeed, the SPELL program identified 7 potential split sites (I64/Q65, G99/E100, Q113/A114, A146/I147, D167/K168, N232/L233, and E256/G257) that are suitable for splitting (Figure 2B). The predicted values of three parameters, including loop position, solvent accessible area (saa), and sequence conservation (cons), and split energy were shown in Supplementary Table 1. The split energy is used to assess the physical scoring function (Dagliyan et al., 2018). A successful split site is always away from major split energy minima (the core labeled as “©” in Figure 2C). D167/K168 pointed out by orange line was close to the energy minima (Figure 2C). It implies that D167/K168 is probably not a good candidate for obtaining a successful split site. An ideal split site should also be located in the evolutionarily non-conserved loop and exposed at surface (Dagliyan et al., 2018). We observed that (1) A146/I147 is not located in the loop and not exposed at surface; (2) Q113/A114 and D167/K168 are relatively conserved during evolution (Supplementary Table 1). According to SPELL prediction results, Q113/A114, A146/I147, and D167/K168 may not be good candidates to make split TurboID.

To evaluate these 7 potential split sites, we next utilized confocal imaging to monitor the subcellular localization of the designed constructs. We attached the N-terminal part of split-TurboID to STIM1, thereby anchoring the fusion protein to the cytosolic side of ER membrane. The C terminal region of split TurboID was fused with SspB and tagged with mCherry (SspB-TurboID(C)-mCherry). In order to achieve a nearly 1:1 expression ratio of both parts, we connected these split fusion proteins with a P2A self-cleaving peptide sequence (Figures 2D,E, upper panel) (He et al., 2021). We found that split TurboID variants at Q113/A114, A146/I147, or D167/K168 sites showed spontaneous ER distribution even before blue light stimulation, indicating a tendency to pre-associate without light stimulation. The variant inserted at N232/L233 displayed an even distribution even after blue light stimulation (Figure 2E, lower panel). These results indicated that these four sites might not be suitable for designing a light-switchable split TurboID system.

Encouragingly, the three variants with the optical dimerizer inserted at I64/Q65, G99/E100, and E256/G257 exhibited light-dependent recruitment of the C-terminal part toward ER-anchored N-terminal fragment (Figure 2E). The site I64/Q65 was the previously reported miniTurbo truncated site, in which the first 64 residues of TurboID was truncated but still retained high labeling activity (Branon et al., 2018); and the E256/G257 site has been previously reported (Schopp et al., 2017). Therefore, we omitted these two sites and focused on the G99/E100 site to develop OptoID.

We next asked whether the biotinylation activity can be restored after light-induced reconstitution of split-TurboID. HeLa cells were transfected with STIM1-OptoID-mCherry, and then incubated with biotin either in the dark or under blue light stimulation for 1 h. Indeed, in the presence of biotin, the variant with the optical dimerizer inserted at positions G99/E100 (thereafter named as OptoID) displayed light-dependent biotinylation, as evidenced by positive staining of biotinylated proteins surrounding the ER network in fixed cells with a biotin antibody conjugated to a green fluorophore (Figure 2F). Collectively, these findings established the feasibility of using OptoID to conditionally catalyze biotinylation in a light-dependent fashion.

### The Biotinylation Kinetics of OptoID

**FIGURE 3:**
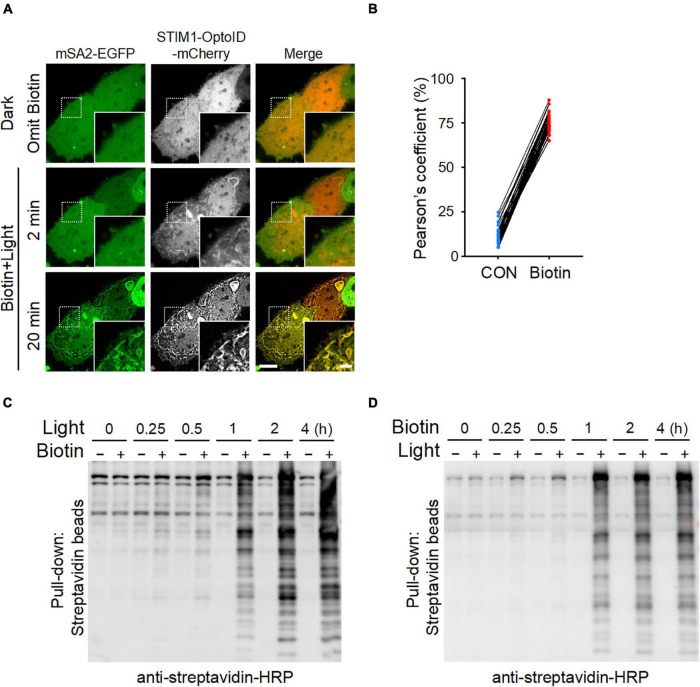
The biotinylation kinetics of OptoID. Photostimulation was applied at 470 nm at a power density of 4 mW/cm^2^ (1 s ON for each 5 s). **(A)** Confocal images of HeLa cells co-expressing STIM1-OptoID-mCherry and mSA2-EGFP before and after photostimulation in response to biotin treatment (100 μM). Scale bar, 10 μm. The mCherry-channel images are shown in grayscale. Also see Supplementary Movie 2. **(B)** Pearson’s correlation coefficient for colocalization between mSA2-EGFP and STIM1-OptoID-mCherry was calculated from **(A)** (*n* = 30 cells from three biological replicates). **(C,D)** Blots of lysates of HeLa cells transiently transfected with STIM1-OptoID-mCherry in response to blue light at the indicated hours and treated with or without biotin (100 μM, **C**). The cells were treated with biotin at the indicated hours in the dark or under blue light stimulation **(D)**. Biotinylation was analyzed by using streptavidin-HRP.

We further applied the optimized mSA-EGFP (mSA2-EGFP) biosensor to monitor the kinetics of OptoID-mediated biotinylation at real time in living cells. We co-expressed mSA2-EGFP with STIM1-OptoID-mCherry in HeLa cells and observed the changes in subcellular localization of mSA2-EGFP. Time-lapsed imaging showed that the biosensor mSA2-EGFP evenly distributed in the cytosol before blue light illumination. Following blue light illumination, the C-terminal fragment of OptoID, indicated by mCherry fluorescence, was rapidly recruited toward the ER within 2 min (Figure 3A and Supplementary Movie 2). Within 20 min, we observed a gradual accumulation of mSA2-EGFP from the cytosol to the ER membrane in the presence of biotin, with consequent colocalization of mSA2-EGFP with ER-bound STIM1-OptoID-mCherry (Figures 3A,B). As a stringent control, mSA2-EGFP was still evenly distributed in the cytosol under the same photostimulation condition in the absence of biotin treatment (Supplementary Figure 2A). We also found that the Pearson’s coefficient value was reduced almost by four times (43.03 ± 6.57% for STIM1-TurboID-mCherry vs. 12.23 ± 4.74% for STIM1-OptoID-mCherry) before biotin treatment, but retained its activity in response to biotin treatment (74.83 ± 5.20% for STIM1-TurboID-mCherry vs. 74.38 ± 5.57% for STIM1-OptoID-mCherry) (Figures 1C, 3B). In parallel, we took a biochemical approach to independently assess light-induced promiscuous biotinylation (Figures 3C,D). HeLa cells transfected with STIM1-OptoID-mCherry were illuminated with pulsed blue light in the presence or absence of biotin (Figure 3C), or treated with biotin at different time points in the dark or under blue light stimulation (Figure 3D). Cell lysates were extracted and protein samples were separated on sodium dodecyl sulfate polyacrylamide gel electrophoresis (SDS-PAGE), followed by immunoblotting with an HRP-conjugated streptavidin to capture and visualize biotinylated proteins. At 15 min, we started to notice appreciable biotinylation, with the degree of biotinylation gradually increased over the time course of 4 h (Figures 3C,D). No further biotinylation was observed in response to a pulse of 15 min light stimulation and switching off the light for 4 h in the presence of biotin (Supplementary Figure 2B). The biotinylation activity is dramatically increased upon continuous light stimulation in the present of biotin (Figure 3D). Together, these observations suggested that the OptoID system has the advantages of low background and high biotinylation efficiency comparable to TurboID.

Blue light typically penetrates human skin to a depth of less than 1–2 mm, which significantly limits the application of optogenetic tools *in vivo* (Tan et al., 2017; Nguyen et al., 2020). To circumvent this roadblock and enable more extensive split-TurboID applications, we further explored a chemical-inducible dimerization approach to reconstitute split-TurboID. To do this, we replaced the iLID system in OptoID with the FKBP12-T2A-FRB pair, in which the dimerization can be induced by the addition of rapamycin (Putyrski and Schultz, 2012), termed ChemoID (DeRose et al., 2013; Lee et al., 2017). Similar to the behavior of STIM1-OptoID-mCherry, ER translocation of STIM1-ChemoID-mCherry was substantially increased upon rapamycin addition (Supplementary Figure 3A). We also observed that the biotinylation biosensor mSA2-EGFP translocated toward the cytosolic side of ER membrane after rapamycin and biotin incubation, suggesting the successful development of a chemical inducible biotinylation system (Supplementary Figures 3B,C).

## Discussion and Conclusion

In the present study, we described a photo-switchable version of split-TurboID, designated OptoID, that can be conditionally assembled by using light. We have demonstrated that the light-dependent reconstitution of OptoID is rapid and maintains efficient catalytic activity when reassembled. Aside from optogenetic approaches, the chemical-inducible dimerization (CID) techniques have been applied to trigger protein-protein interactions upon the addition of small molecules (Jing et al., 2020). In particular, the FKBP and FRB domain-based CID system is most widely used to manipulate biomolecular activities (Castellano and Chavrier, 2000; Castellano et al., 2000; Jing et al., 2020). Considerable efforts have been devoted to generate split BioID/TurboID system (Table 1), aiming to reduce the basal background biotinylation activity seen in the full length BioID or TurboID. In these designs, the inactive N/C terminal halves of BioID/TurboID are individually fused to FRB or FKBP to form a rapamycin-induced functional biotin ligase. According to structural analysis on BirA (PDB ID: 1BIB), Schopp et al. (2017) designed Split-BioID by using the site E256/G257. In parallel, Kwak et al. (2020) identified splitting sites based on temperature factors (B factors), and used the G78/G79 site to reconstitute biotinylation activity upon rapamycin treatment. In addition, Cho et al. (2020) used split sites predicted by the SPELL algorithm based on two BirA structures (PDB ID: 1HXD and 2EWN) (Weaver et al., 2001; Wood et al., 2006). They found that TurboID with the split site at L73/G74, named as Split-TurboID, displayed the best rapamycin-dependent activity (Cho et al., 2020). In addition to the FKBP-FRB system (De Munter et al., 2017), fused the two inactive halves of BirA* to a protein phosphatase PP1 and PP1-interacting proteins (PIPs), respectively. Upon heterodimerization of the phosphatase subunits when they are closely positioned in space, inactive halves of BirA* form a functional biotin ligase (De Munter et al., 2017). This method was used to sensitively report protein-protein interaction. These systems have two notable problems. First, rapamycin is an inhibitor of mTORC1, which is critical for multiple cellular functions, such as cell growth, proliferation, and selective autophagy (Wullschleger et al., 2006; Zoncu et al., 2011). Therefore, rapamycin may have imposed potential side effects to its native signaling component, thereby confounding data interpretation when used *in cellulo* or *in vivo* (Zoncu et al., 2011). Second, chemical-induced dimerization or chemogenetic manipulation is known to lack strict spatial control, thereby hampering their applications.

To enable both spatial and temporal control over TurboID activity and obviate the need of small molecules, we replaced the chemical-inducible system with an optical dimerizer to restore TurboID activity upon photostimulation. We applied the same SPELL algorithm to predict potential split sites in BirA, but we used the structure of PBD ID: 1BIB (Wilson et al., 1992). Up to 6 top potential sites, I64/Q65, G99/E100, Q113/A114, A146/I147, D167/K168, and N232/L233, were selected for experiment testing (Figures 2B–E). We found that three split variants, Q113/A114, A146/I147, and D167/K168, showed spontaneous reconstitution even before blue light stimulation (Figure 2E, lower panel); while the variant N232/L233 showed no light-induced changes in biotinylation (Figure 2E, lower panel), making them not ideal for designing a light-switchable split TurboID system. Furthermore, I64/Q65 exhibited light-dependent assembly, but the previously developed miniTurbo is truncated at this position and retains high biotinylation activity (Branon et al., 2018). Although E256/G257 showed light-dependent enzymatic activity, this site has been previously used to develop Split-BioID (Schopp et al., 2017). Therefore, we focused our study on using a totally new split site situated at G99/E100, which displayed high biotinylation activity after blue light stimulation (Figures 2F, 3). A side-by-side comparison among these tools was summarized in Table 1.

In parallel, we have optimized an EGFP-tagged monomeric streptavidin (mSA2) to serve as a biotinylation biosensor, which permits real-time monitoring of protein biotinylation in living cells (Figure 3). OptoID offers several advantages over TurboID. First, a functional TurboID is only generated upon light-induced heterodimerization of the split fragments, thereby eliminating the background biotinylation issue associated with conventional TurboID. Second, by harnessing the power of light, we achieved precise spatial and temporal control of TruboID activity. Hence, we expect OptoID described herein could be a novel tool to study the components of protein complexes in living cells. OptoID will also be a valuable tool for the otherwise challenging study such as probing organelle contact sites, thereby contributing to the discovery of high-resolution protein interactome maps in spatiotemporal precision.

## Data Availability Statement

The raw data supporting the conclusions of this article will be made available by the authors, without undue reservation.

## Author Contributions

YZ and JJ conceived the ideas and directed the work and designed the study. RC and NZ performed the experiments and analyzed the results. NZ contributed to the discussion and editing of the manuscript. RC, YZ, and JJ wrote the manuscript. All authors contributed to the discussion and editing of the manuscript.

## Conflict of Interest

The authors declare that the research was conducted in the absence of any commercial or financial relationships that could be construed as a potential conflict of interest.

## Publisher’s Note

All claims expressed in this article are solely those of the authors and do not necessarily represent those of their affiliated organizations, or those of the publisher, the editors and the reviewers. Any product that may be evaluated in this article, or claim that may be made by its manufacturer, is not guaranteed or endorsed by the publisher.
