# Supplementary material for: Optical Sensors and Actuators for Probing Proximity-Dependent Biotinylation in Living Cells
Source: Front Cell Neurosci. 2022 Feb 16;16:801644. doi: 10.3389/fncel.2022.801644 (PMC8890125; doi:10.3389/fncel.2022.801644)
Supplement: Supplementary file 1 [file Data_Sheet_1.PDF]

Supplementary materials include:

Captions for Supplementary Movies

Captions for Supplementary Table 1

Supplementary Figures 1-3

Supplementary Movie 1| Time-lapse confocal imaging of HeLa cells co-expressing STIM1-TurboID mCherry and mSA2-EGFP in response to biotin treatment (100 μM) for 10 min. Scale bar, 10 μm.

Supplementary Movie 2| Time-lapse confocal imaging of HeLa cells co-expressing STIM1-OptoID-mCherry and mSA2-EGFP upon exposure to pulsed 488-nm confocal laser stimulation (1 s ON for each 5 s) and biotin treatment (100μM) for 20 min. Scale bar, 10 μm.

Supplementary Table 1| Raw date of split energy profile and three parameters (Loop, solvent accessible area (saa), and sequence conservation (cons)). Split sites were highlighted in red

Figure S1.

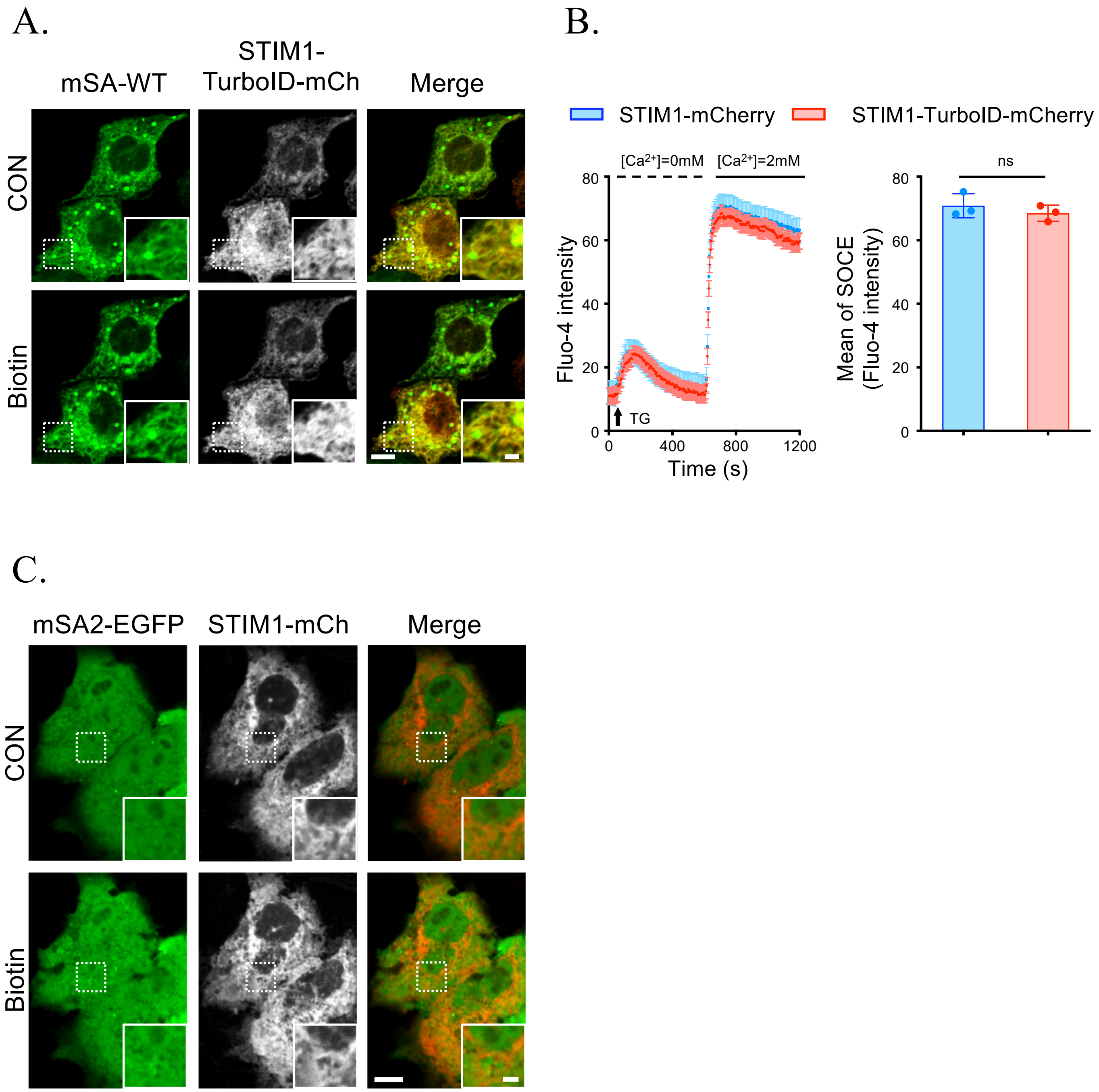

Figure S1 | High background biotinylation activity of TurboID

(A) Confocal images of HeLa cells expressing mSA-WT-EGFP and STIM1-TurboID-mCherry with or without biotin treatment (100 μM). Scale bar, 10 μm. The insets show the boxed regions outlined at higher magnification. Scale bar, 2 μm. The mCherry-channel images are shown in greyscale.
(B) Representative recordings of Ca^{2+} influx as indicated by Fluo-4 in HeLa cells overexpressed STIM1-mCherry or STIM1-TurboID-mCherry (left panel). Quantitation of SOCE was shown on the right. Data were shown as mean ± sd (ns, not significant; SOCE, store-operated Ca^{2+} entry; TG, thapsigargin).
(C) HeLa cells were expressed with mSA2-EGFP and STIM1-mCherry and treated with biotin for 20 min(100 μM). Scale bar, 10 μm. High-magnification images of boxed areas are shown at the bottom right. Scale bar, 2 μm. The mCherry-channel images are shown in greyscale.

Figure S2.

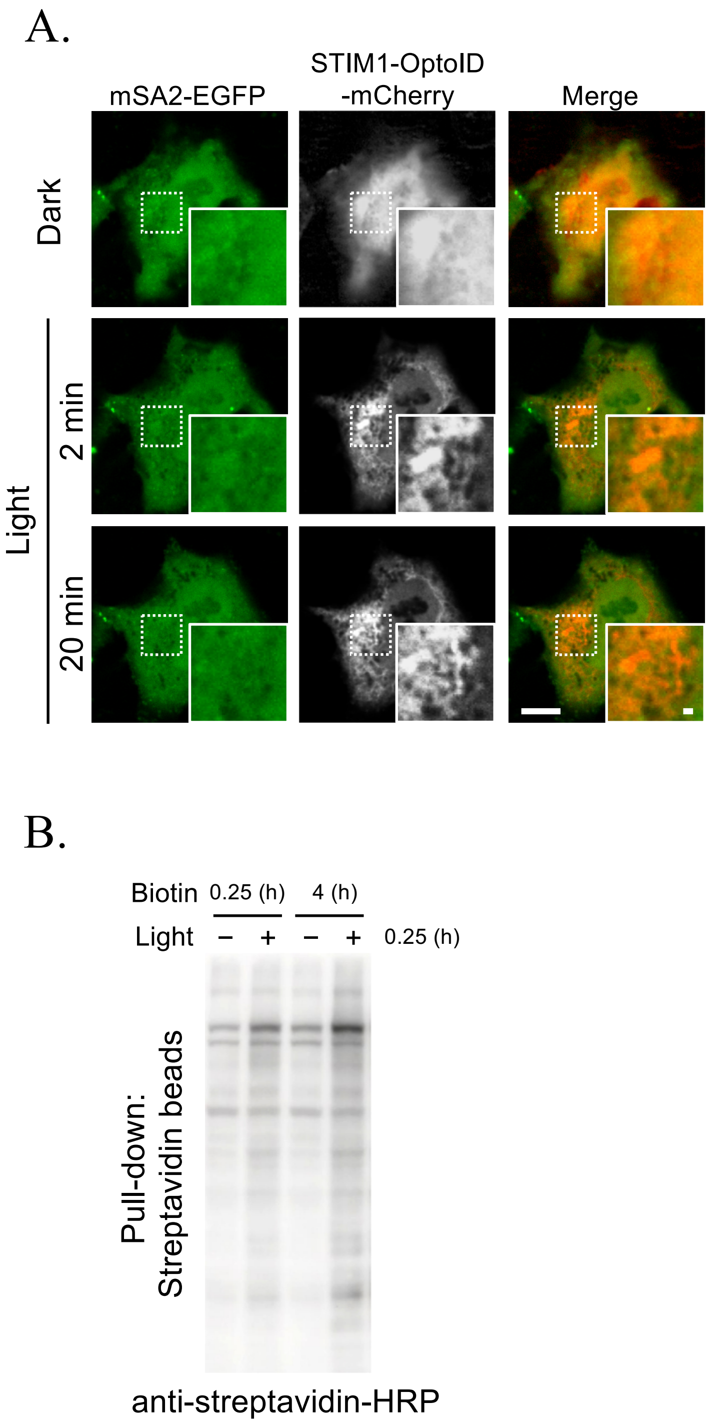

Figure S2 |

(A) HeLa cells were expressed with mSA2-EGFP and STIM1-TurboID-mCherry and illuminated with blue light for 20 min. Scale bar, 10 µm. The insets show the boxed regions outlined at higher magnification. Scale bar, 2 µm. The mCherry-channel images are shown in greyscale.

(B) Blots of lysates of HeLa cells transiently transfected with STIM1-OptoID-mCherry in response to blue light for 0.25 hours with or without Biotin (line 1 and 2); or switch off the light after a pulse of 0.25 hours illumination and harvest the cells after 4h biotin treatment (line 3 and 4). Biotinylation was analyzed by using streptavidin-HRP.

Figure S3.

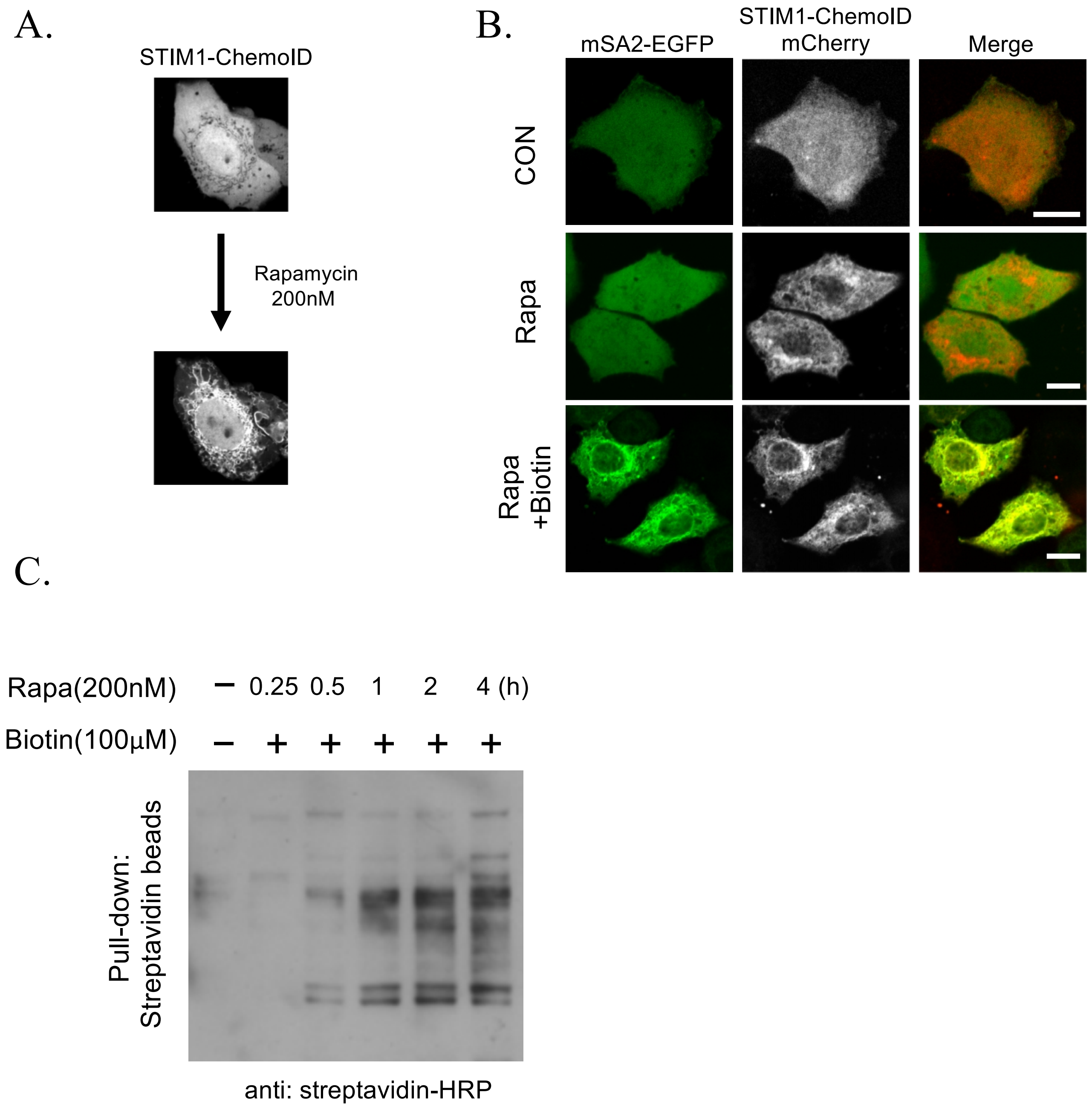

Figure S3 | Chemical controllable reassembly of split TurboID. The mCherry-channel images are shown in greyscale.

(A) Confocal images of HeLa cells expressing STIM1-TurboID(N)-FRB-T2A-FKBP-TurboID(C)-mCherry (FRB-T2A-FKBP was inserted at G99/E100, STIM1-ChemoID-mCherry) before and after rapamycin treatment (200nM). Scale bar, 10 μm.

(B) Confocal images of HeLa cells co-expressing STIM1-ChemoID-mCherry and mSA2-EGFP with or without rapamycin treatment (200nM). in response to biotin treatment (100μM). Scale bar, 10 μm. Rapa, Rapamycin.

(C) Immunoblot analysis on lysates of HeLa cells transiently transfected with STIM1-ChemoID-mCherry in response to rapamycin treatment at the indicated hours (treated with or without biotin). Biotinylation was visualized by using streptavidin-HRP.
